# The outcomes of right-sided and left-sided colonic diverticulitis following non-operative management: a systematic review and meta-analysis

**DOI:** 10.1186/s13017-022-00463-7

**Published:** 2022-11-01

**Authors:** Sih-Shiang Huang, Chih-Wei Sung, Hsiu-Po Wang, Wan-Ching Lien

**Affiliations:** 1 Department of Emergency Medicine, National Taiwan University Hospital, Taipei, Taiwan; 2 Department of Emergency Medicine, National Taiwan University Hsin-Chu Hospital, Hsinchu City, Taiwan; 3 Department of Internal Medicine, National Taiwan University Hospital, Taipei, Taiwan; 4 Department of Internal Medicine, College of Medicine, National Taiwan University, Taipei, Taiwan; 5 Department of Emergency Medicine, National Taiwan University College of Medicine, National Taiwan University, No.7, Chung-Shan South Road, Taipei, 100 Taiwan

**Keywords:** Colonic diverticulitis, Right-sided, Left-sided, Non-operative management, Recurrence, Treatment failure

## Abstract

**Background:**

There is no sufficient overview of outcomes in right-sided and left-sided colonic diverticulitis (CD) following non-operative management. This systematic review was conducted to evaluate the recurrence/treatment failure in right-sided and left-sided CD.

**Methods:**

A systematic review was conducted following PRISMA guidelines. MEDLINE, Embase, and Cochrane Library from inception to Dec 2021 were searched. The study characteristics, recurrence/treatment failure, and risk factors for recurrence/treatment failure were extracted. Proportional meta-analyses were performed to calculate the pooled recurrent/treatment failure rate of right-sided and left-sided CD using the random effect model. Logistic regression was applied for the factors associated with the recurrence/treatment failure.

**Results:**

Thirty-eight studies with 10,129 patients were included, and only two studies comprised both sides of CD. None of the studies had a high risk of bias although significant heterogeneity existed. The pooled recurrence rate was 10% (95% CI 8–13%, I^2^ = 86%, *p* < 0.01) in right-sided and 20% (95% CI 16–24%, I^2^ = 92%, p < 0.01) in left-sided CD. For the uncomplicated CD, the pooled recurrence rate was 9% (95% CI 6–13%, I^2^ = 77%, *p* < 0.01) in right-sided and 15% (95% CI 8–27%, I^2^ = 97%, *p* < 0.01) in the left-sided. Age and gender were not associated with the recurrence of both sides. The treatment failure rate was 5% (95% CI 2–10%, I^2^ = 84%, *p* < 0.01) in right-sided and 4% (95% CI 2–7%, I^2^ = 80%, *p* < 0.01) in left-sided CD. The risk factors for recurrence and treatment failure were limited.

**Conclusion:**

Non-operative management is effective with low rates of recurrence and treatment failure for both right-sided and left-sided CD although left-sided exhibits a higher recurrence. The recurrence rates did not differ between patients receiving antibiotics or not in uncomplicated CD. Age and sex were not associated with the recurrence although other risk factors were dispersing. Further risk factors for recurrence and treatment failure would be investigated for precise clinical decision-making and individualized strategy.

## Introduction

Acute colonic diverticulitis (CD), referring to the inflammation of the diverticula, is one of the common diagnoses for patients with abdominal pain [1]. The incidence of CD has been increasing over time, i.e. the percentage of acute admissions was increasing by 16% in males and 12% in females during 10 years in England [2] and a 26% increase in presentations from 1998 to 2005 in the United States [3]. Although CD exhibits a low mortality rate, it would lead to enormous morbidity including pelvic abscess, intestinal perforation, bowel fistula, bowel obstruction, peritonitis, or sepsis [4]. A substantial percentage of recurrence occurs following an acute episode of CD despite complete remission [5, 6]. Also, certain patients experienced treatment failure and proceeded to surgery.

The characteristics of right-sided CD differ from left-sided in many respects. Most of the right-sided diverticula are solitary, containing all bowel layers (true diverticula). Right-sided CD occurs more frequently in Asians with fewer complications [7–9]. By contrast, left-sided CD occurs mainly in the western population. However, there is no sufficient overview of recurrence/treatment failure in the right-sided and left-sided CD in the literature [10]. Therefore, this systematic review was conducted to evaluate the recurrence/treatment failure in the right-sided and left-sided CD following non-operative management.

## Materials and methods

The systematic review was performed based on Preferred Reporting Items for Systematic Reviews and Meta-Analyses (PRISMA) guidelines [11]. The review protocol was registered in PROSPERO (CRD42021278200). Ethical committee review was waived at the study institution.

### Data resources and study selection

We performed a literature review in 3 databases, MEDLINE, Embase, and Cochrane Library, searching for relevant studies from inception to Dec 2021. We used boolean combinations of keywords including “diverticulitis” or “diverticulosis” combined with “recurrent” or “recurrence” or “relapsed” or “location”. Our search was limited to the English language only. Two authors (Huang SS and Sung CW) reviewed the titles and abstracts to extract eligible studies, which were those that reported recurrent rates of left-sided or right-sided diverticulitis. A manual search of the reference lists of the included articles was also conducted to include other relevant studies. Also, we excluded articles that did not record the locations of the diverticulitis. A third author (Lien WC) made the final decision regarding discrepancies. We excluded case reports, case series, duplicate articles, editorials, and studies involving surgical interventions.

### Data extraction and quality assessment

Full-text articles were screened for eligible studies reporting the recurrent rates of left-sided or right-sided CD. We also extracted the population number, baseline characteristics, severity score of diverticulitis, duration of antibiotic treatment, percutaneous drainage, length of hospital stay, length of follow-up time, and mortality. Two authors (Huang SS and Sung CW) performed quality assessments using the revised Cochrane tool for assessing the risk of bias in randomized trials (RoB 2 tool) [12] and the Newcastle–Ottawa Scale (NOS) [13]. The RoB 2 tool is one of the most commonly used tools for risk of bias assessment in randomized trials. The NOS is a standard tool for quality assessment of nonrandomized studies in systematic review and meta-analysis.

### Data analysis

To calculate the pooled recurrence/treatment failure rates of right-sided and left-sided diverticulitis, proportional meta-analyses were performed. The pooled recurrence and treatment failure rates were presented with proportions and 95% confidence intervals (CIs). The Dersimonian-Laird random effects model with Hartung-Knapp variance correction was applied to improve the estimation [14, 15]. Forest plots were drawn to inspect the results visually and heterogeneity across the included studies was estimated with the Higgins statistics (I^2^) [16]. Logistic regression was applied for the factors associated with the recurrence/treatment failure. All analyses were performed using R 4.1.1 software (R Foundation for Statistical Computing, Vienna, Austria). A *p*-value of < 0.05 was considered statistically significant.

## Results

### Article selection, characteristics, and quality of the included studies

**Fig. 1:**
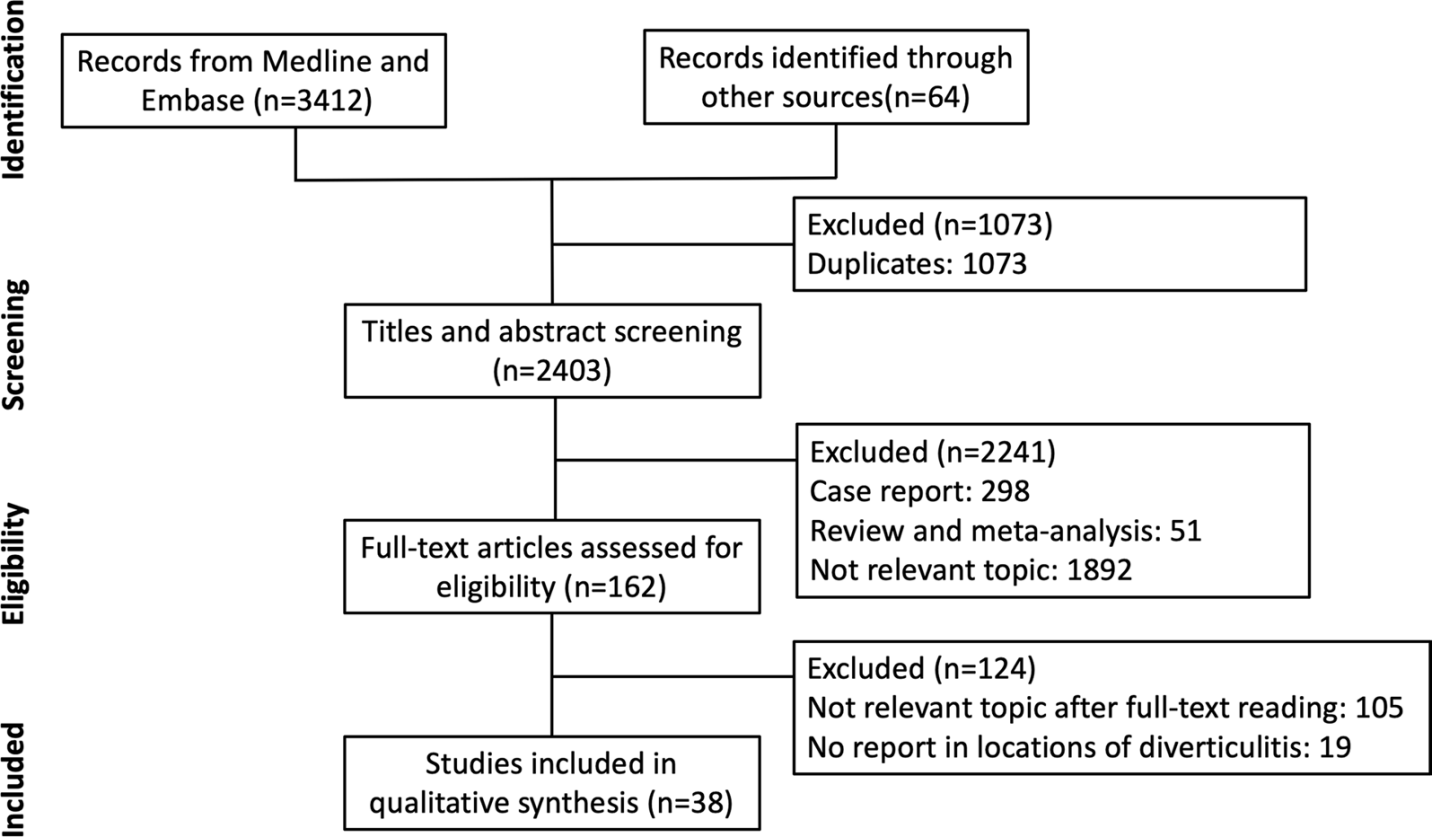
Evidence search and selection

The initial search identified 3,412 studies from MEDLINE and Embase and 64 studies from manual search (Fig. 1). After removing 1,073 duplicates, 2,403 articles were screened through the titles and abstract; 2,241 articles were excluded after initial screening and the remaining 162 studies were retrieved for full-text review. Finally, 38 studies with 10,129 patients were included in this study, and 2 studies comprised both sides of CD [17, 18].

**Fig. 2:**
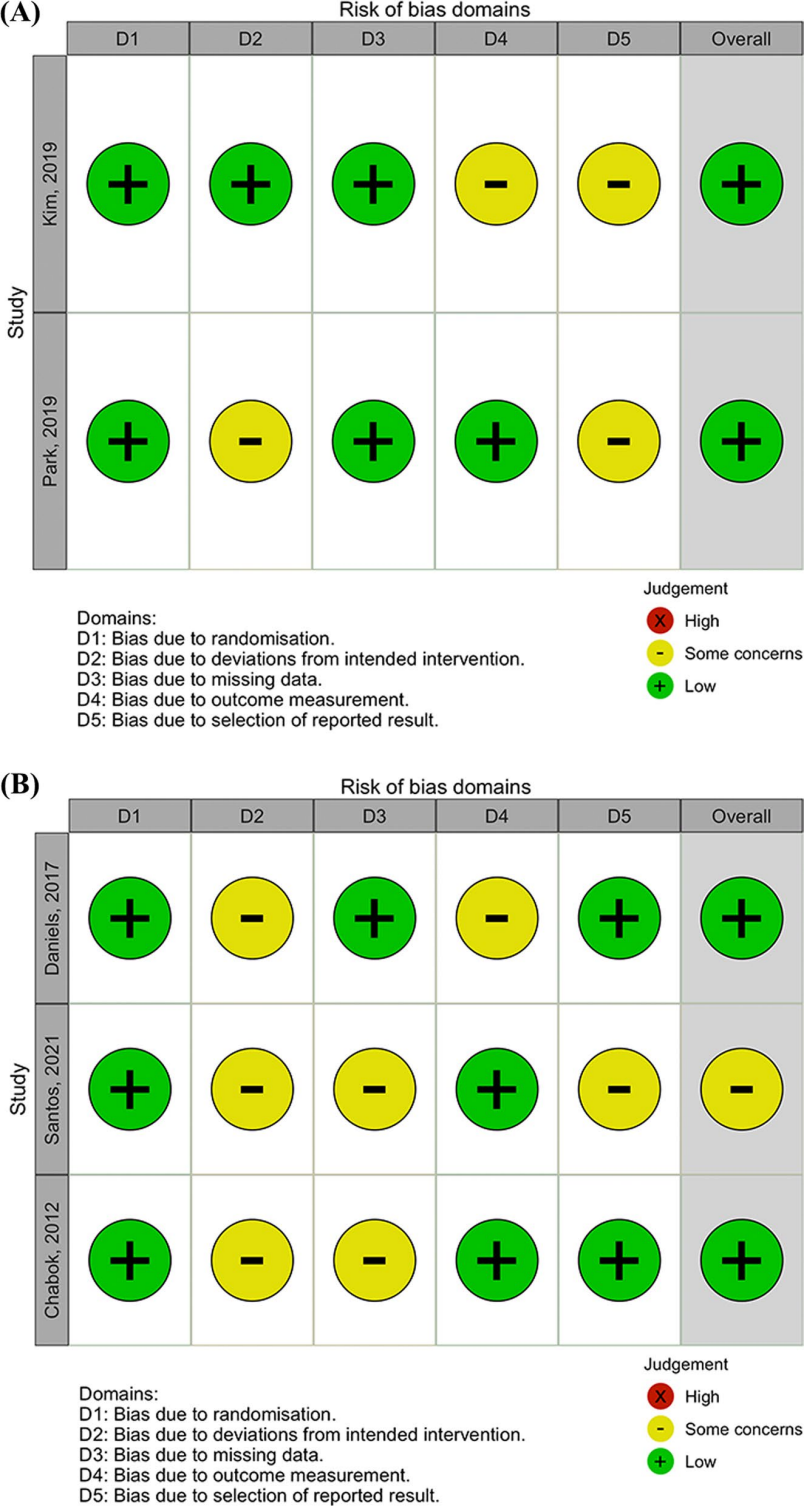
The risk of bias using the revised Cochrane tool for assessing the risk of bias in randomized trials (RoB 2 tool). **A** Right-sided colonic diverticulitis. **B** Left-sided colonic diverticulitis

**Fig. 3:**
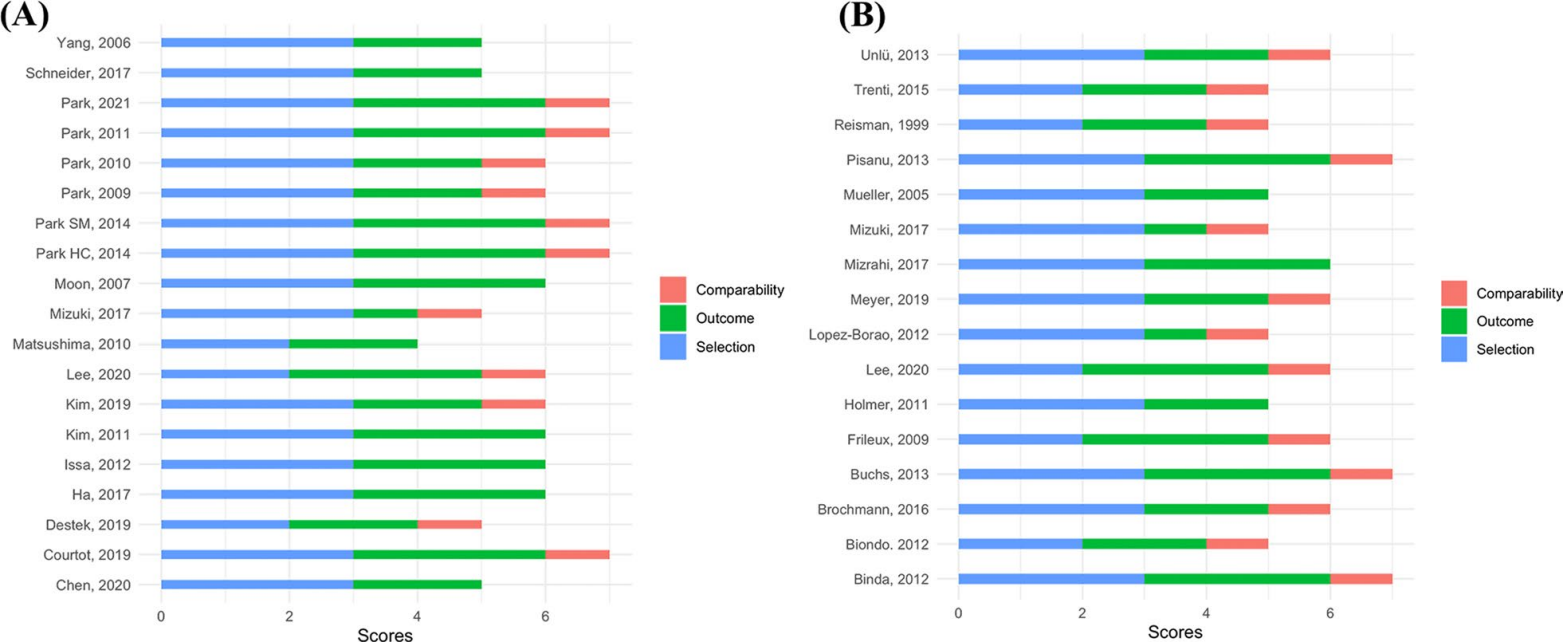
The risk of bias using the Newcastle–Ottawa Scale (NOS) for quality assessment of nonrandomized studies. **A** Right-sided colonic diverticulitis. **B** Left-sided colonic diverticulitis

Twenty-one studies investigated the recurrence of right-sided CD and 19 studies were for left-sided CD. The detailed information was listed in Table 1. Two randomized clinical trials for the right side and 3 for the left side were evaluated by the RoB 2 tool and no high risk of potential bias was detected (Fig. 2). Nineteen cohort studies for the right side and 16 for the left side were assessed by the NOS and none of the studies had a high risk of bias (Fig. 3).

**Table 1:** The detailed information of the included studies

Author	Design^*^	period	brief conclusion
Right-sided diverticulitis			
Mizuki et al. [17]	RCS	1998–2009	The rate of recurrence did not differ between left-sided and right-sided diverticulitis (20% vs 27%, *p* = NS^*^)
Chen et al. [21]	RCS	2013–2020	Right-sided diverticulitis with non-operative treatment had a low recurrence rate (2%)
Moon et al. [22]	RCS	2001–2005	Conservative treatment should be considered a safe and effective option for acute right-sided diverticulitis as its low rate of recurrence (3.6%) and treatment failure (0%)
Issa et al. [23]	RCS	2005–2007	The current study supports conservative therapy for right-sided diverticulitis due to a low recurrence rate (7%) and treatment failure rate (0%)
Ha et al. [24]	RCS	2005–2012	Conservative treatment had a low recurrence rate (10%) in patients with acute right-sided diverticulitis
Park et al. [25]	RCS	1998–2007	Conservative treatment is primarily required for right-sided diverticulitis, and the recurrence rate was 8%. The rate of treatment failure was 1%
Kim et al. [26]	RCS	2008–2009	Conservative treatment with antibiotics should be the treatment of choice for right-sided diverticulitis which had a recurrence rate of 13%
Park et al. [27]	PCS	2007–2009	Outpatient management for uncomplicated right-sided diverticulitis had a similar recurrence rate compared with inpatient management (10% vs 11%, *p* = NS^*^). No treatment failure was noted in both groups
Park et al. [20]	PCS	2004–2012	The recurrence rate for medical treatment of right-sided diverticulitis was 19%; of those, diverticula located only in the right colon and a single diverticulum had a lower recurrence rate
Park et al. [19]	RCS	2017–2019	In medical treatment for right-sided diverticulitis, 9% ended up with treatment failure. Elderly (Age > 50), recurrent episodes, and elevated C-reactive protein levels are factors associated with treatment failure. The recurrence rate was 13% in the conservative treatment success group
Kim et al. [28]	RCT	2014–2018	The overall recurrence rate of medical treatment for right-sided diverticulitis was 9%. No significant differences were shown between the antibiotics and the non-antibiotics group in the recurrence rate (10% vs 8%, *p* = NS^*^) and the treatment failure rate (2% vs. 5%, *p* = NS^*^)
Kim et al. [8]	RCS	2001–2014	The recurrence rate was 11% in right-sided diverticulitis. Smoking and longer hospital stay were the risk factors for recurrence
Park et al. [9]	RCS	2002–2012	16% of the included patients had a recurrence of right-sided diverticulitis. Multiple diverticula and intraperitoneally located diverticulitis may have a higher recurrence rate
Courtot et al. [29]	RCS	2003–2017	The article consisted of western populations, right-sided diverticulitis had a recurrence rate of 10.4% and treatment failure rate of 6%
Schneider et al. [30]	RCS	2005–2013	The recurrence rate of right-sided diverticulitis was 16.7%. In Caucasians, right-sided diverticulitis occurred in younger and thinner patients compared to left-sided
Destek et al. [31]	RCS	2014–2017	The overall rate of recurrence was 21.1% in acute right-sided diverticulitis
Lee et al. [18]	RCS	2011–2017	The recurrence rate of left-sided diverticulitis was higher than right-sided (19.1% vs 6.9%, *p* = 0.003). In addition, compared to left-sided, right-sided diverticulitis presented as younger age and less advanced modified Hinchey stages
Matsushima et al. [32]	RCS	1994–2005	Conservative therapy had a low recurrence rate of 8.4% in acute right-sided diverticulitis
Park et al. [33]	RCS	2000–2007	The overall recurrence rate was 2%. However, patients who had a pericolic abscess showed a high recurrence rate of 20%
Park et al. [34]	RCT	2011–2014	No significant differences were shown between one-day versus four-day antibiotics treatment strategy in recurrence (10.3% vs 9.0%, *p* = NS^*^) and treatment failure (17.2% vs 21.3%, *p* = NS^*^)
Yang et al. [35]	RCS	1994–2004	The recurrence rate of acute right-sided diverticulitis was 12.6%. For recurrent diseases, conservative treatment was still safe and efficient
Left-sided diverticulitis			
Daniels et al. [36]	RCT	2010–2014	The recurrence rate was low (3% vs 3%, *p* = NS^*^) in both strategies with or without antibiotics within 6 months of follow-up. The rate of treatment failure was 7% and 11% in the antibiotics group and observation group respectively
Pisanu et al. [8]	RCS	2006–2011	The rate of recurrence was similar (6% vs 20%, *p* = NS^*^) between the older and younger groups. No patient suffered from treatment failure under medical treatment in this study
Mizuki et al. [17]	RCS	1998–2009	The rate of recurrence did not differ between left-sided and right-sided diverticulitis (20% vs 27%, *p* = NS^*^)
Santos et al. [37]	RCT	2018–2020	Conservative treatment of complicated sigmoid diverticulitis had a high recurrence rate (27%) within 6 months
Holmer et al. [38]	PCS	2004–2007	The rate of treatment failure was low (4%). However, conservative treatment had a much higher recurrence than the surgical intervention (33% vs 4%, *p* < 0.001)
Mizrahi et al. [39]	RCS	1998–2008	Conservative treatment for sigmoid diverticulitis had a recurrence rate of 21%, but no emergent surgery was required during long-term follow-up
Chabok et al. [40]	RCT	2003–2010	The rate of recurrence did not differ between antibiotics and non-antibiotics groups (16% vs 16%, *p* = NS^*^) in the treatment of left-sided diverticulitis. Also, the rate of treatment failure was similar between antibiotics and non-antibiotics groups (1% vs. 0.3%, *p* = NS^*^)
Reisman et al. [42]	RCS	1987–1997	The rate of recurrence was 30%. No significant difference in recurrence was found between older (> 60 years) and younger (≤ 60 years) patients, whether treated conservatively or surgically
Muller et al. [41]	RCS	1985–1991	Although the recurrence rate of sigmoid diverticulitis was reported high (47%), lethal complications were still rare. Also, the rate of treatment failure was 0%
Frileux et al. [43]	RCS	1995–2002	The recurrence rate of sigmoid diverticulitis was high (43%), but recurrence attacks were rarely severe. Operative treatment was not advised after the first attack of diverticulitis
Lopez-Borao et al. [44]	RCS	1998–2008	The overall recurrence rate of diverticulitis was 20.5%. The rate of treatment failure was 6.9%. Younger age (< 45 years) was not a risk of recurrence
Binda et al. [45]	RCS	1996–1999	The rate of recurrence was higher in medical treatment than in operative treatment (17.2% vs 5.8%, *p* < 0.001). Risk factors for recurrence included medical treatment, younger age (< 40 years), and a history of at least 3 episodes of acute diverticulitis. In addition, the overall treatment failure rate was 6.9%
Biondo et al. [46]	RCS	1994–2008	The rate of recurrence was 20.7%. No significant differences were shown between the immunosuppressed patients and the nonimmunosuppressed patients (21.5% vs 20.5%, *p* = NS^*^)
Unlü et al. [47]	RCS	2004–2012	The recurrence rate of acute sigmoid diverticulitis was 24.4%. The younger group (≤ 50 years) was not associated with a higher recurrence rate than the older group (25.6% vs 23.8%, *p* = NS^*^)
Buchs et al. [48]	PCS	2007–2011	The overall recurrence rate was 16.4% in uncomplicated sigmoid diverticulitis. A high level of serum C-reactive protein (> 240 mg/L) was associated with early recurrence (< 6 months) (*p* = NS^*^)
Trenti et al. [49]	RCS	1994–2011	The recurrence rate of acute diverticulitis was 14.8% in this study. The univariate analysis showed higher recurrence in patients under chronic corticoid therapy (*p* = 0.043) and the presence of more than 1 abscess (*p* < 0.001)
Brochmann et al. [50]	RCS	2013–2014	The recurrence rate of uncomplicated left-sided diverticulitis was 6.7%, and the treatment failure rate was 3.6%. No significant differences were shown between the patients treated with antibiotics and those without antibiotics (10.9% vs 4.6%, *p* = NS^*^)
Lee et al. [18]	RCS	2011–2017	The recurrence rate of left-sided diverticulitis was higher than right-sided (19.1% vs 6.9%, *p* = 0.003). In addition, compared to left-sided, right-sided diverticulitis presented as younger age and less advanced modified Hinchey stages
Meyer et al. [51]	RCS	2005–2009	In Hinchey 1a diverticulitis, the overall recurrence rate was 23.9%. The presence of extraluminal air on computed tomography did not show a higher recurrence rate (17.9% vs 25.3%, *p* = NS^*^)

^*^RCS Retrospective cohort study, *PCS* Prospective cohort study, *RCT* Randomized clinical trial, *NS* Non-significant

### Right-sided CD

A total of 3,931 patients were included and most of them were from the eastern population [8, 9, 17–35]. The mean age was 41.4 years, with 57% being males. The median follow-up period ranged from 4 to 90 months and it was lacking in 3 studies [19, 25, 32]. Notably, only 4 studies were conducted outside of eastern Asia [23, 29–31]. Seventeen studies came from eastern Asia (13 from South Korea, 2 from Japan, 1 from China, and 1 from Taiwan), and the mean age ranged from 35 to 46 years.

Ten studies reported the details of antibiotic treatment: 7 with 2nd or 3rd generation of cephalosporin plus metronidazole [19, 23, 27, 28, 31, 33, 34] and 3 with adding aminoglycoside [22, 26, 36]. The duration ranged from 1 to 14 days. Twenty-seven (0.7%) patients received percutaneous drainage.

**Fig. 4:**
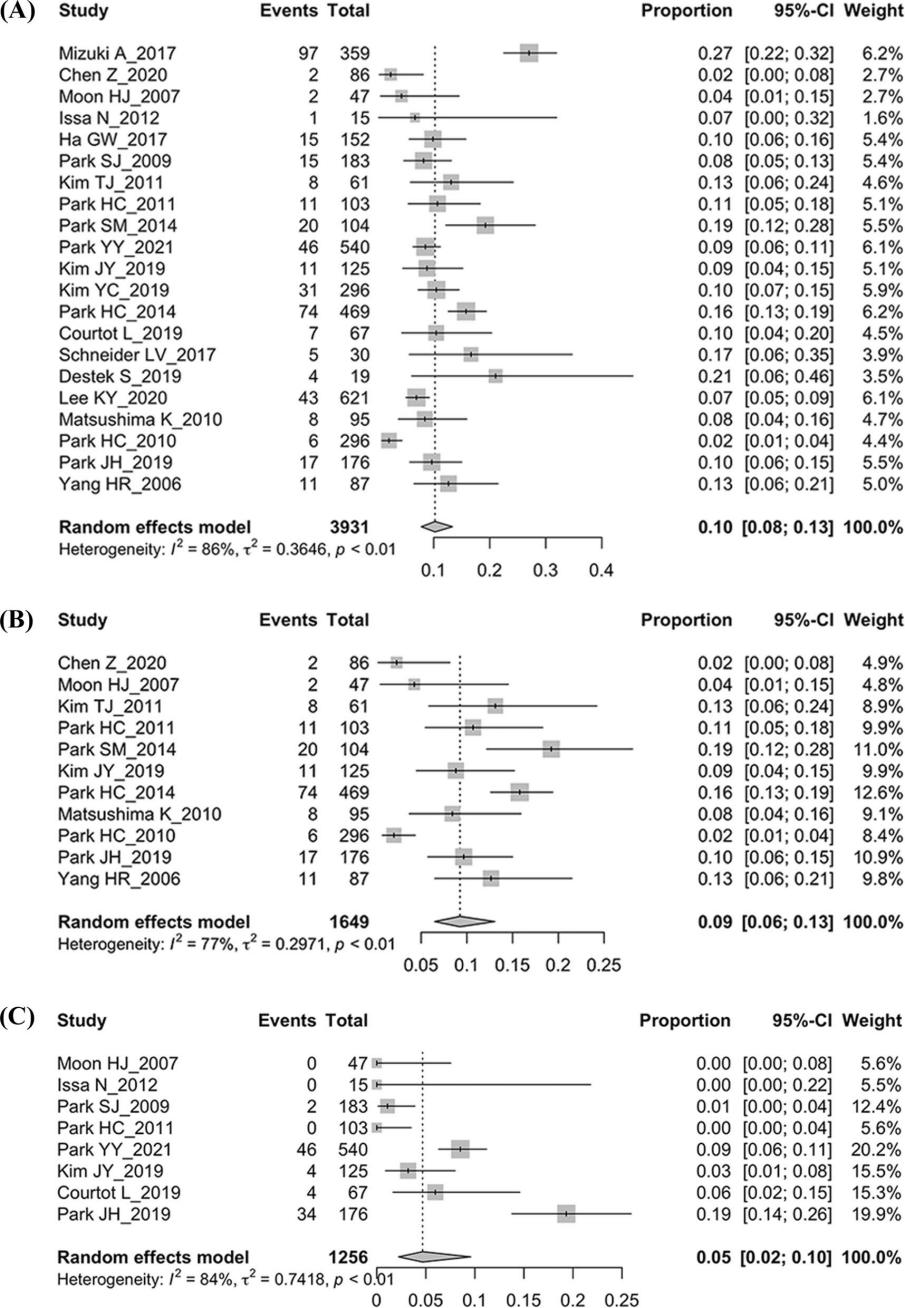
The forest plot of right-sided diverticulitis. **A** Recurrence. **B** Recurrence of uncomplicated diverticulitis. **C** Treatment failure

We used the random effect model for analysis and the pooled recurrence rate was 10% (95% CI 8–13%) (Fig. 4A). Subgroup analysis showed a recurrence rate of 9% (95% CI 6–13%) within the uncomplicated diverticulitis (Fig. 4B). High heterogeneity was observed between the studies. We also performed a subgroup analysis investigating the relationship between the use of antibiotics and recurrence. The result demonstrated no significant difference in recurrence rates between the antibiotics and antibiotics-free group (9% vs. 8%, *p* = 0.85).

Further analysis demonstrated that age (*p* = 0.184) and gender (*p* = 0.932) were not related to the recurrence. The reported factors associated with recurrence were limited. Park et al. reported that patients with multiple diverticula outside the right-sided colon were associated with a higher risk of recurrence [20]. Kim et al. presented that smoking and longer hospital stay may be the risk factors for recurrence [8].

Also, 8 of the 21 included studies reported the rate of treatment failure, and the pooled results showed a failure rate of 5% (95% CI 2–10%) (Fig. 4C). Patients would proceed to surgical intervention following treatment failure. The risk factors related to failure in non-operative management were limited. Park et al. reported that elderly (age > 50 years), recurrent episodes, and elevated C-reactive protein levels were risk factors of failure in conservative treatment [19]. Multiple diverticula were also related to the treatment failure [34].

### Left-sided CD

A total of 6,198 patients were included, and most of them were from the western population [17, 18, 36–52]. The mean age was 61.4 years and 45% were males. The median follow-up period ranged from 6 to 128 months.

Only 4 studies reported the regimen of antibiotic treatment: one with cephalosporin plus metronidazole for at least 7 days [42], one with ampicillin/sulbactam for at least 7 days [40], one with amoxicillin/clavulanic acid for 10 days [37], one with ampicillin, metronidazole, and aminoglycoside for 5 days [36]. Twenty-six (0.4%) patients underwent percutaneous drainage.

The recurrence rates in the included studies were varying. In patients with sigmoid diverticulitis, Mueller et al. reported the highest recurrence rate of 47% (78/167), and 17% (13/78) of them required surgery [43]. Frileux et al. reported a recurrence rate of 43% (55/128) [44] and Holmer et al. reported 33% (13/40) [40]. By contrast, several studies reported successful results with recurrence rates of 3–6% following conservative treatment [37, 38, 51].

**Fig. 5:**
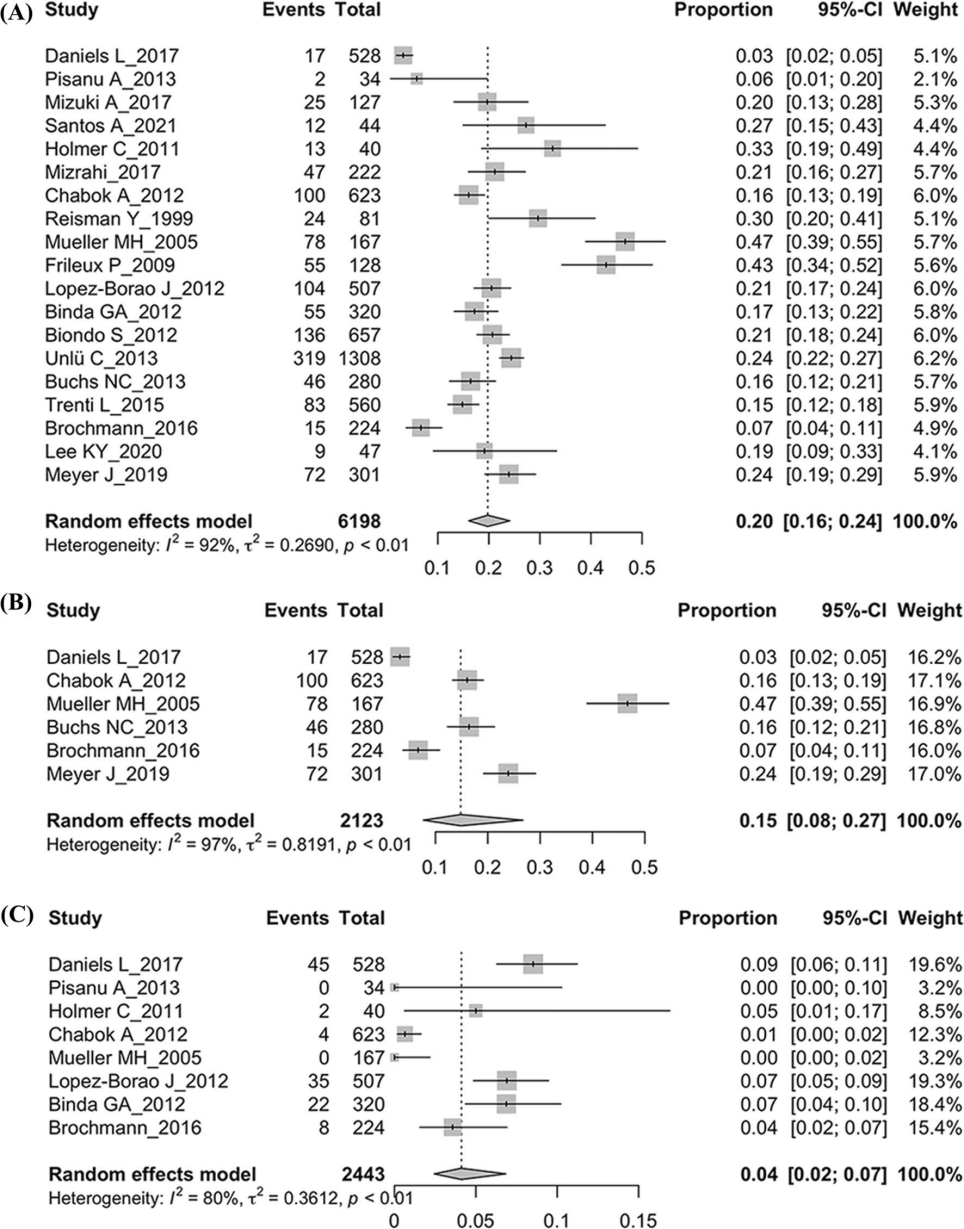
The forest plot of left-sided diverticulitis. **A** Recurrence. **B** Recurrence of uncomplicated diverticulitis. **C** Treatment failure

The pooled recurrence rate was 20% (95% CI 16–24%) using the random effect model (Fig. 5A) and the recurrence rate of uncomplicated CD was 15% (95% CI 8–27%) (Fig. 5B). High heterogeneity existed between the included studies. Moreover, we investigate the effects of antibiotics-free on recurrence. The pooled results showed similar recurrence rates (20% vs. 19%, *p* = 0.334) in patients receiving antibiotics or not.

The regression analysis regarding the effects of age and gender on the recurrence was performed. The results showed age (*p* = 0.587) and gender (*p* = 0.988) were not related to the recurrence. Holmer et al. reported that previous recurrent episodes of sigmoid diverticulitis, perforated sigmoid diverticulitis, and conservative treatment were risk factors for recurrent sigmoid diverticulitis [21]. Trenti et al. reported the use of steroids and more than one abscess were associated with recurrence [50].

Eight of the 19 included studies reported the rate of treatment failure, and the pooled results showed a failure rate of 4% (95% CI 2–7%) (Fig. 5C). Patients would proceed to surgery. No other specific risk factors for treatment failure were reported in the included studies.

## Discussion

We performed a systematic review and meta-analysis to investigate the recurrence/treatment failure of right-sided and left-sided CD following non-operative management. Thirty-eight comparative studies with a total of 10,129 patients were identified. To the best of our knowledge, this is the largest meta-analysis currently.

Our analyses showed that non-operative management was a safe first-line treatment for CDs with low rates of recurrence and treatment failure. The recurrence rate of left-sided CD was significantly higher than that of right-sided CD (20% vs. 10%). There was no significant difference in the recurrence rates of uncomplicated CD (9% vs. 15%) and treatment failure rates (5% vs. 4%) between right-sided and left-sided CD, manifesting as overlapping in the 95% CI of the pooled data. The pooled results of age, sex, and the use of antibiotics were not related to the recurrence. The other risk factors for recurrence and treatment failure were diverse and limited in the included studies. None of the included studies had a high risk of bias although heterogeneity existed.

Non-operative management includes antibiotic treatment and percutaneous drainage. In this review, the details and duration of antibiotic treatment were not discussed because the data was diverse or lacking. Few patients received percutaneous drainage which indicated the effectiveness of antibiotic treatment only.

In the included studies, a significantly higher recurrence rate was reported among patients with sigmoid diverticulitis [36, 39, 40, 43, 44, 48]. The pooled recurrence rate of left-sided CD would be skewed. The results implied that the sigmoid colon was the most commonly affected location of recurrence, as reported by Sung et al. [52]. However, the locations could not be investigated in this work because most of the studies did not report the exact locations of the CD but only classified it as a left-sided or right-sided CD.

Left-sided CD exhibited a higher recurrence rate than right-sided although there was no difference in the uncomplicated CD. It could be speculated that more recurrences occurred in the left-sided complicated CD. However, the data regarding complicated CD was limited in the included studies so further investigation will be needed.

Although the patients with right-sided CD were younger and had male predominance in our study, as those in a current meta-analysis [10], age and sex were not associated with the recurrence. The risk factors for recurrence were reported in 9 included studies [8, 9, 19, 20, 33, 46, 49, 50], however, the evidence was scarce and disperse. Radiographic characteristics (e.g. multiple diverticula, more than one abscess), previous recurrent episodes, laboratory data (e.g. C-reactive protein), smoking, long hospital stay, and the use of steroids were noted across different studies.

Moreover, the evidence regarding treatment failure was also limited. One study reported that elderly (age > 50 years), recurrent episodes, and elevated C-reactive protein levels were risk factors for failure in conservative treatment [24]. The other study reported multiple diverticula were associated with treatment failure [34].

Further, recent meta-analyses concluded no beneficial impact for antibiotic treatment in the CD [53, 54]. Our results showed the use of antibiotics was not associated with recurrence in both left-sided and right-sided CD. However, all of the studies regarding antibiotic-free treatment included patients with uncomplicated CD only. The interpretation should be cautious and more evidence is needed to investigate the efficacy of antibiotic treatment in CD.

This study has several limitations. First, the included studies were highly heterogeneous with clinical inconsistency. Also, selection bias would exist due to the retrospective nature in more than half of the studies (29/38). However, our study is by far the most comprehensive systematic review regarding the recurrence/treatment failure of left-sided and right-sided CD. None of the included studies had a high risk of bias. Second, the majority of studies regarding left-sided CDs were conducted in western countries. By contrast, most of the studies discussing right-sided CD were from the Asian population. The results would be extrapolated cautiously to Asian patients with the left-sided CD or vice versa. Third, the details of comorbidities and severity scores for recurrence and treatment failure were lacking across the studies; thus, risk factors of recurrence and treatment failure other than age and sex cannot be thoroughly analyzed. Further studies would focus on the risk factors of recurrence and treatment failure and make an individualized treatment strategy.

## Conclusion

Non-operative management is effective for right-sided and left-sided CDs with low rates of recurrence and treatment failure although left-sided exhibits a higher rate of recurrence. The recurrence rates did not differ between patients receiving antibiotics or not although current evidence focused on uncomplicated CD. Age and sex were not associated with the recurrence although other risk factors were dispersing. Further investigations should be needed for risk factors for precise clinical decision-making and individualized strategy.

## Data Availability

All data analyzed during this study are included in this published article.

## Abbreviations

CD: Colonic diverticulitis
PRISMA: Preferred reporting items for systematic reviews and meta-analyses
RoB 2 tool: Revised cochrane tool for assessing the risk of bias in randomized trials
NOS: Newcastle–Ottawa scale
CI: Confidence interval

## References

[1] Meltzer AC, Pines JM, Richards LM, Mullins P, Mazer-Amirshahi M (2017). US emergency department visits for adults with abdominal and pelvic pain (2007–13): trends in demographics, resource utilization and medication usage. Am J Emerg Med.

[2] Kang JY, Hoare J, Tinto A, Subramanian S, Ellis C, Majeed A (2003). Diverticular disease of the colon–on the rise: a study of hospital admissions in England between 1989/1990 and 1999/2000. Aliment Pharmacol Ther.

[3] Etzioni DA, Mack TM, Beart RW, Kaiser AM (2009). Diverticulitis in the United States: 1998–2005: changing patterns of disease and treatment. Ann Surg.

[4] Theodoropoulos D (2018). Current options for the emergency management of diverticular disease and options to reduce the need for colostomy. Clin Colon Rectal Surg.

[5] Hupfeld L, Burcharth J, Pommergaard H-C, Rosenberg J (2017). Risk factors for recurrence after acute colonic diverticulitis: a systematic review. Int J Colorectal Dis.

[6] Ho VP, Nash GM, Milsom JW, Lee SW (2015). Identification of diverticulitis patients at high risk for recurrence and poor outcomes. J Trauma Acute Care Surg.

[7] Tan K-K, Wong J, Yan Z, Chong C-S, Liu JZ, Sim R (2014). Colonic diverticulitis in young Asians: a predominantly mild and right-sided disease. ANZ J Surg.

[8] Kim Y-C, Chung J-W, Baek J-H, Lee W-S, Kim D, Park Y-H (2019). Risk factors for recurrence of right colonic diverticulitis. Dig Surg.

[9] Park H-C, Kim BS, Lee K, Kim MJ, Lee BH (2014). Risk factors for recurrence of right colonic uncomplicated diverticulitis after first attack. Int J Colorectal Dis.

[10] Hajibandeh S, Hajibandeh S, Smart NJ, Maw A (2020). Meta-analysis of the demographic and prognostic significance of right-sided versus left-sided acute diverticulitis. Colorectal Dis.

[11] Page MJ, McKenzie JE, Bossuyt PM, Boutron I, Hoffmann TC, Mulrow CD (2021). The PRISMA 2020 statement: an updated guideline for reporting systematic reviews. BMJ.

[12] Sterne JAC, Savović J, Page MJ, Elbers RG, Blencowe NS, Boutron I (2019). RoB 2: a revised tool for assessing risk of bias in randomised trials. BMJ.

[13] Stang A (2010). Critical evaluation of the Newcastle-Ottawa scale for the assessment of the quality of nonrandomized studies in meta-analyses. Eur J Epidemiol.

[14] DerSimonian R, Kacker R (2007). Random-effects model for meta-analysis of clinical trials: an update. Contemp Clin Trials.

[15] Jackson D, Law M, Rücker G, Schwarzer G (2017). The Hartung-Knapp modification for random-effects meta-analysis: a useful refinement but are there any residual concerns?. Stat Med.

[16] Higgins JPT, Thompson SG (2002). Quantifying heterogeneity in a meta-analysis. Stat Med.

[17] Mizuki A, Tatemichi M, Nakazawa A, Tsukada N, Nagata H, Kanai T (2017). Changes in the clinical features and long-term outcomes of colonic diverticulitis in japanese patients. Intern Med.

[18] Lee KY, Lee J, Park YY, Kim Y, Oh ST (2020). Difference in clinical features between right- and left-sided acute colonic diverticulitis. Sci Rep.

[19] Park YY, Nam S, Han JH, Lee J, Cheong C (2021). Predictive factors for conservative treatment failure of right colonic diverticulitis. Ann Surg Treat Res.

[20] Park SM, Kwon TS, Kim DJ, Lee YS, Cheung DY, Oh ST (2014). Prediction and management of recurrent right colon diverticulitis. Int J Colorectal Dis.

[21] Chen Z, Zhang B, Wu D, Jin Y (2020). Characteristics of predominantly right-sided colonic diverticulitis without need for colectomy. BMC Surg.

[22] Moon HJ, Park JK, Lee JI, Lee JH, Shin HJ, Kim WS (2007). Conservative treatment for patients with acute right colonic diverticulitis. Am Surg.

[23] Issa N, Paran H, Yasin M, Neufeld D (2012). Conservative treatment of right-sided colonic diverticulitis. Eur J Gastroenterol Hepatol.

[24] Ha GW, Lee MR, Kim JH (2017). Efficacy of conservative management in patients with right colonic diverticulitis. ANZ J Surg.

[25] Park SJ, Choi SI, Lee SH, Lee KY (2009). Image-guided conservative management of right colonic diverticulitis. World J Gastroenterol.

[26] Kim TJ, Lee IK, Park JK, Lee YS, Si Y, Jung H (2011). Is conservative treatment with antibiotics the correct strategy for management of right colonic diverticulitis?: a prospective study. J Korean Soc Coloproctol.

[27] Park H-C, Kim BS, Lee BH (2011). Management of right colonic uncomplicated diverticulitis: outpatient versus inpatient management. World J Surg.

[28] Kim JY, Park SG, Kang HJ, Lim YA, Pak KH, Yoo T (2019). Prospective randomized clinical trial of uncomplicated right-sided colonic diverticulitis: antibiotics versus no antibiotics. Int J Colorectal Dis.

[29] Courtot L, Bridoux V, Lakkis Z, Piessen G, Manceau G, Mulliri A (2019). Long-term outcome and management of right colonic diverticulitis in western countries: multicentric retrospective study. J Visc Surg.

[30] Schneider LV, Millet I, Boulay-Coletta I, Taourel P, Loriau J, Zins M (2017). Right colonic diverticulitis in caucasians: presentation and outcomes versus left-sided disease. Abdom Radiol (NY).

[31] Destek S, Gul VO (2019). Effectiveness of conservative approach in right colon diverticulitis. Ulus Travma Acil Cerrahi Derg.

[32] Matsushima K (2010). Management of right-sided diverticulitis: a retrospective review from a hospital in Japan. Surg Today.

[33] Park HC, Chang MY, Lee BH (2010). Nonoperative management of right colonic diverticulitis using radiologic evaluation. Colorectal Dis.

[34] Park JH, Park HC, Lee BH (2019). One-day versus four-day antibiotic treatment for acute right colonic uncomplicated diverticulitis: a randomized clinical trial. Turk J Gastroenterol.

[35] Yang HR, Huang HH, Wang YC, Hsieh CH, Chung PK, Jeng LB (2006). Management of right colon diverticulitis: a 10-year experience. World J Surg.

[36] Reisman Y, Ziv Y, Kravrovitc D, Negri M, Wolloch Y, Halevy A (1999). Diverticulitis: the effect of age and location on the course of disease. Int J Colorectal Dis.

[37] Daniels L, Ünlü Ç, de Korte N, van Dieren S, Stockmann HB, Vrouenraets BC (2017). Randomized clinical trial of observational versus antibiotic treatment for a first episode of CT-proven uncomplicated acute diverticulitis. Br J Surg.

[38] Pisanu A, Vacca V, Reccia I, Podda M, Uccheddu A (2013). Acute diverticulitis in the young: the same disease in a different patient. Gastroenterol Res Pract.

[39] Santos A, Mentula P, Pinta T, Ismail S, Rautio T, Juusela R (2021). Comparing laparoscopic elective sigmoid resection with conservative treatment in improving quality of life of patients with diverticulitis: the laparoscopic elective sigmoid resection following diverticulitis (LASER) randomized clinical trial. JAMA Surg.

[40] Holmer C, Lehmann KS, Engelmann S, Gröne J, Buhr HJ, Ritz J-P (2011). Long-term outcome after conservative and surgical treatment of acute sigmoid diverticulitis. Langenbecks Arch Surg.

[41] Mizrahi I, Al-Kurd A, Chapchay K, Ag-Rejuan Y, Simanovsky N, Eid A (2018). Long-term outcomes of sigmoid diverticulitis: a single-center experience. J Surg Res.

[42] Chabok A, Påhlman L, Hjern F, Haapaniemi S, Smedh K, Group AS (2012). Randomized clinical trial of antibiotics in acute uncomplicated diverticulitis. Br J Surg.

[43] Mueller MH, Glatzle J, Kasparek MS, Becker HD, Jehle EC, Zittel TT (2005). Long-term outcome of conservative treatment in patients with diverticulitis of the sigmoid colon. Eur J Gastroenterol Hepatol.

[44] Frileux P, Dubrez J, Burdy G, Roullet-Audy JC, Dalban-Sillas B, Bonnaventure F (2010). Sigmoid diverticulitis. Longitudinal analysis of 222 patients with a minimal follow up of 5 years. Colorectal Dis.

[45] Lopez-Borao J, Kreisler E, Millan M, Trenti L, Jaurrieta E, Rodriguez-Moranta F (2012). Impact of age on recurrence and severity of left colonic diverticulitis. Colorectal Dis.

[46] Binda GA, Arezzo A, Serventi A, Bonelli L (2012). Italian study group on complicated D, Facchini M, et al multicentre observational study of the natural history of left-sided acute diverticulitis. Br J Surg.

[47] Biondo S, Borao JL, Kreisler E, Golda T, Millan M, Frago R (2012). Recurrence and virulence of colonic diverticulitis in immunocompromised patients. Am J Surg.

[48] Unlu C, van de Wall BJ, Gerhards MF, Wiezer M, Draaisma WA, Consten EC (2013). Influence of age on clinical outcome of acute diverticulitis. J Gastrointest Surg.

[49] Buchs NC, Konrad-Mugnier B, Jannot AS, Poletti PA, Ambrosetti P, Gervaz P (2013). Assessment of recurrence and complications following uncomplicated diverticulitis. Br J Surg.

[50] Trenti L, Kreisler E, Galvez A, Golda T, Frago R, Biondo S (2015). Long-term evolution of acute colonic diverticulitis after successful medical treatment. World J Surg.

[51] Brochmann ND, Schultz JK, Jakobsen GS, Oresland T (2016). Management of acute uncomplicated diverticulitis without antibiotics: a single-centre cohort study. Colorectal Dis.

[52] Meyer J, Caruso A, Roos E, Balaphas A, Toso C, Poletti PA (2019). The clinical significance of extraluminal air in Hinchey 1a diverticulitis: results from a retrospective cohort study with 10-year follow-up. Int J Colorectal Dis.

[53] Balk EM, Adam GP, Bhuma MR, Konnyu KJ, Saldanha IJ, Beland MD (2022). Diagnostic imaging and medical management of acute left-sided colonic diverticulitis: a systematic review. Ann Intern Med.

[54] Desai M, Fathallah J, Nutalapati V, Saligram S (2019). Antibiotics versus no antibiotics for acute uncomplicated diverticulitis: a systematic review and meta-analysis. Dis Colon Rectum.

